# Sleep-Disordered Breathing—Do We Have to Change Gears in Heart Failure?

**DOI:** 10.1007/s11897-016-0304-x

**Published:** 2016-09-17

**Authors:** Martin R. Cowie

**Affiliations:** National Heart and Lung Institute, Imperial College London (Royal Brompton Hospital), Dovehouse Street, London, SW3 6LY UK

**Keywords:** Heart failure, Sleep-disordered breathing, Sleep apnoea

## Abstract

The majority of patients with heart failure have sleep-disordered breathing (SDB)—with central (rather than obstructive) sleep apnoea becoming the predominant form in those with more severe disease. Cyclical apnoeas and hypopnoeas are associated with sleep disturbance, hypoxaemia, haemodynamic changes, and sympathetic activation. Such patients have a worse prognosis than those without SDB. Mask-based therapies of positive airway pressure targeted at SDB can improve measures of sleep quality and partially normalise the sleep and respiratory physiology, but recent randomised trials of cardiovascular outcomes in central sleep apnoea have been neutral or suggested the possibility of harm, likely from increased sudden death. Further randomised outcome studies (with cardiovascular mortality and hospitalisation endpoints) are required to determine whether mask-based treatment for SDB is appropriate for patients with chronic systolic heart failure and obstructive sleep apnoea, for those with heart failure with preserved ejection fraction, and for those with decompensated heart failure. New therapies for sleep apnoea—such as implantable phrenic nerve stimulators—also require robust assessment. No longer can the surrogate endpoints of improvement in respiratory and sleep metrics be taken as adequate therapeutic outcome measures in patients with heart failure and sleep apnoea.

## Introduction

Sleep-disordered breathing (SDB), or sleep apnoea, is common in patients with cardiovascular disease, and its presence is associated with a poorer prognosis and high healthcare costs [1, 2]. International guidelines suggest that it is worthwhile to screen for this condition. Recent evidence suggests that the relationship between SDB and the underlying cardiovascular condition may be complex, particularly in heart failure (HF). Although there is a strong therapeutic rationale for the treatment of daytime sleepiness due to obstructive sleep apnoea in the non-heart failure population, the possibility exists that central sleep apnoea may be at least partially adaptive in HF patients and treating this may be harmful in some circumstances. The results of recent randomised trials are challenging our current understanding of the pathophysiology of SDB and the effects of currently available therapies on clinical outcome.

## What Is Sleep-Disordered Breathing?

**Fig. 1:**
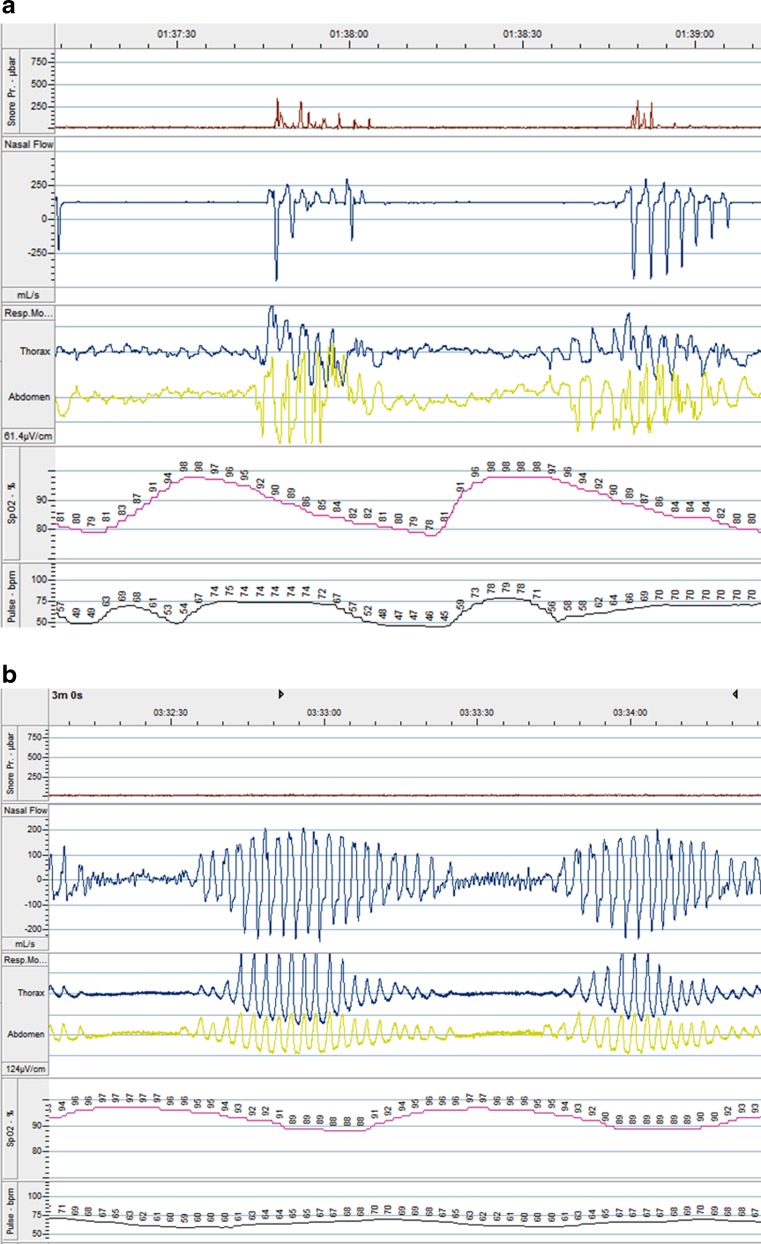
Polygraph recordings from a patient with **a** OSA and **b** CSA. Note the continuation of respiratory movement during the period of apnoea in OSA, but the absence of respiratory effort during apnoea in CSA. *First panel* is noise related to snoring (seen in **a** not **b**), *second* is nasal air flow, *third* is thoracic and abdominal wall movement, *fourth* is arterial oxygen saturation, and *fifth* is pulse rate (modified from reference [15])

The two major phenotypes of SDB are obstructive sleep apnoea (OSA) and central sleep apnoea (CSA) (Fig. 1). In OSA (the most common form of SDB in the general population), there is collapse of the pharynx during sleep with consequent upper airway obstruction, often with snoring [3]. Predisposing factors include obesity, a short neck, and retrognathism. Rostral fluid shift during sleep in HF can lead to pharyngeal edema, which may exacerbate the tendency to obstruction [4]. CSA, the other type of SDB, is usually associated with heart failure, although it has also been observed in patients with stroke, especially in the acute phase, and in those with renal failure or opiate use. In CSA, the underlying abnormality is in the regulation of breathing in the respiratory centres of the brainstem, with a marked reduction or cessation of respiratory effort. Patients with HF and CSA tend to have an exaggerated respiratory response to CO_2_, associated with excess sympathetic nervous activity and increased chemosensitivity. A modest rise in PaCO_2_ during sleep results in inappropriate hyperventilation [5–7], driving PaCO_2_ below the apnoeic threshold, at which point the neural drive to respire is too low to stimulate effective inspiration and an apnoea (complete pause in breathing) or hypopnoea (partial reduction in airflow) ensues. PaCO_2_ subsequently rises and the cycle is repeated. This overshoot of the homeostatic feedback loop is exacerbated by the prolonged circulation time between the alveoli and the brainstem seen in more severe HF. In addition, pulmonary congestion stimulates J receptors in the lungs, triggering reflex hyperventilation. A particular form of CSA is a periodic pattern of hyperventilation followed by hypoventilation, termed Cheyne-Stokes respiration (CSR). CSR is not limited to sleep but can occur at rest, or during exercise, in patients with advanced HF [8, 9].

A tendency to progress from OSA to CSA over the course of the night has been observed in HF. This is thought to be secondary to progressive pulmonary congestion and deteriorating hemodynamics [10].

Recently, it has been suggested that CSR (although a marker of a poor prognosis) may be a compensatory mechanism in patients with heart failure [11, 12]. Periodic hyperventilation and apnoea may increase end-expiratory lung volume (and therefore oxygen stores), increase vagal tone, aid cardiac pump function, provide intrinsic positive end-expiratory airways pressures, and reduce respiratory muscle fatigue [11].

## How Is SDB Diagnosed and Quantified?

In patients without cardiovascular disease, typical symptoms of SDB include excessive daytime sleepiness, insomnia, morning headaches, depression, cognitive dysfunction, nocturnal dyspnoea, nocturia, and erectile dysfunction. However, there is a wide inter-individual variation in symptoms, especially between male and female patients [13].

Importantly, patients with HF and SDB do not tend to complain of daytime sleepiness, possibly related to high sympathetic tone. Screening questionnaires that include questions about daytime sleepiness (such as the Epworth Sleepiness Scale used to screen for OSA in non-heart failure populations) are therefore not useful [14].

Attended in-hospital polysomnography (PSG), including assessment of respiratory movement, oxygen saturation, nasal and oral airflow, snoring, electroencephalography, electrocardiography, electromyography, and ocular movement, has long been considered the gold standard test for sleep disorders. More limited, multi-channel sleep polygraphy (PG) with oxygen saturation, nasal airflow, and chest and abdominal movement recorded is more widely available and can be set up by the patient at home [15]. Compared with PSG, PG has a sensitivity and specificity of 90–100 % for the diagnosis of significant SDB in patients with HF [16, 17]. Even simpler screening may be performed by recording nocturnal oxygen saturation via a finger probe, with a sensitivity of 93 % and a specificity of 73 % for moderate-to-severe SDB compared to PSG when using a cut-off of 12.5 desaturations of ≥3 % per h for patients: few patients with clinically important SDB would be missed by this simple first-stage approach [18]. Such screening cannot determine the phenotype of SDB, and further investigation with (at least) PG is mandatory in those who test positive and in anyone who tests negative but where clinical suspicion remains high.

The severity of SDB is described by the average number of apnoeic and hypopnoeic events per hour of sleep—the *apnoea-hypopnoea index* (AHI). Apnoea is a reduction in airflow ≥90 % of pre-event baseline for ≥10 s; hypopnoea is a reduction in airflow ≥30 % from baseline for ≥10 s, with a fall in PaCO_2_ ≥3 % or an arousal from sleep [19]. Up to 5 events/h is usually defined as normal, 5–15/h as mild, 15–30/h as moderate, and >30/h as severe SDB. The number and severity of oxygen desaturations may also be used as a metric of the severity of SDB. Additionally, those in whom >50 % of events are obstructive are labelled as *predominantly* OSA, and if >50 % of events are central, such a patient is labelled as predominantly CSA.

Algorithms have been developed in cardiac implantable electronic devices (such as pacemakers and defibrillators) to detect and quantify SDB [20]. The DREAM study reported a sensitivity of 89 % and a specificity of 85 % for the diagnosis of moderate-to-severe SDB by a pacemaker algorithm using transthoracic impedance and minute ventilation sensors [21].

## Risk Factors for SDB in Heart Failure

A recent study of more than 6500 patients in Germany with systolic HF reported a strong association between SDB (either OSA or CSA) and obesity, male sex, atrial fibrillation, age, and poorer left ventricular systolic function [22]. Risk factors for CSA in HF patients referred to a sleep laboratory include male sex (OR = 3.50), atrial fibrillation (OR = 4.13), age >60 years (OR = 2.37), and resting hypocapnia (partial pressure of carbon dioxide (PCO_2_) <38 mmHg during wakefulness; OR = 4.33) [23].

## Physiological Consequences of SDB (Table 1)

**Table 1:** Disease mechanisms linking SDB with heart failure

Sleep apnoea	Intermittent hypoxaemia
	Intermittent hypercapnia
	Increased negative intrathoracic pressure swings
	Increased arousals from sleep
	Sleep deprivation
	Sleep fragmentation
Disease mechanisms	Sympathetic nervous system activation
	Metabolic dysregulation
	Endothelial dysfunction
	Systemic inflammation
	Hypercoagulability
	Impaired cardiac function
	Left atrial enlargement
	Myocardial ischaemia
	Myocardial fibrosis
	Arrhythmia

### Intermittent Hypoxaemia

Cyclical episodes of hypoxaemia-reoxygenation occur in patients with SDB, with increased inflammation and oxygen-derived free radicals analogous to ischaemia/reoxygenation injury [24, 25]. The severity of oxygen desaturation is associated with levels of vascular endothelial growth factor, a stimulator of neo-angiogenesis [26]. Intermittent hypoxaemia and reoxygenation may also result in activation of the pro-inflammatory transcription factor nuclear factor-kB, endothelial cell and leukocyte activation, increased expression of adhesion molecules, and activation of ‘stress’ genes that influence oxygen delivery, such as hypoxia-inducible factor-1 [27–29].

### Sympathetic Nervous System (SNS) Activation

SNS activity is increased in SDB, with both higher muscle SNS activity [30] and elevated urinary norepinephrine concentrations [31], related to upper airway closure, hypoxaemia, hypercarbia, and the arousals associated with the respiratory events. Enhanced SNS activity may stimulate the expression of inflammatory cytokines [32].

### Alterations in Intrathoracic Pressure

The repetitive inspiratory efforts during the apnoeas and hypopnoeas in SDB lead to exaggerated negative intrathoracic pressure swings (up to −65 mmHg intrathoracic pressure in OSA), including increased left ventricular (LV) transmural pressure, increased afterload and right ventricular venous return, and an abnormal leftward shift of the interventricular septum [33]. This leads to increased myocardial oxygen demand, impaired myocardial relaxation, and reduced cardiac output. Progressive increases in intra-atrial pressures lead to atrial myocardial overstretching and dilation, causing cardiac volumetric changes and electrical remodelling that may lead to atrial fibrillation [34]. LV diastolic function declines in association with SDB, especially with ageing [35].

### Cardiac Remodelling

Animal models have shown the development of hypertension, LV hypertrophy, and reduced LV ejection fraction as a result of long-term SDB [36]. In humans, a progressive increase in LV mass index with AHI level, independent of BMI, has been reported in the Sleep Heart Health Study, an observational cross-sectional study investigating cardiovascular outcomes in SDB [37]. More severe SDB, as defined by higher AHI and more hypoxaemia, was associated with greater LV systolic dimensions and lower LV ejection fraction. LV diastolic dysfunction also appears to be poorer in patients with more severe SDB, independent of obesity, diabetes mellitus, and hypertension [38]. SDB may more adversely affect myocardial function in patients with underlying coronary artery disease than in those without [39].

### Sleep Reduction and Fragmentation

SDB exerts its negative physiologic effects in part due to reduced quantity of sleep and excessive sleep fragmentation as a result of repetitive upper airway obstruction-induced sleep disruption. Such sleep deprivation appears to trigger increased inflammation, with elevations in interleukin-6 (IL-6), high-sensitivity C-reactive protein, and leukocyte counts [40, 41].

### Metabolic Dysregulation

Several observational studies have demonstrated associations between sleep apnoea and insulin resistance that are independent of obesity [42], partially mediated by upregulation of inflammatory cytokines [43]. Increased SNS activity associated with SDB may affect glucose homeostasis by increasing glycogen breakdown and gluconeogenesis. Experimental sleep deprivation has been shown to increase evening cortisol concentrations, resulting in pronounced increases in serum glucose levels and insulin concentrations and increased insulin secretion [44].

### Other Abnormalities

Endothelial dysfunction may occur in SDB as a result of systemic inflammation, oxidative stress, and SNS activation. Individuals with SDB have impaired resistance vessel endothelium-dependent dilation [45]. There is some evidence for a hypercoagulable state in sleep apnoea with increased levels of plasminogen activator inhibitor-1 (PAI-1), fibrinogen, activated coagulation factors XIIa and VIIa, thrombin/antithrombin III complexes, and soluble P-selectin [46].

## Pathophysiological Link Between SDB and Heart Failure

The Sleep Heart Health Study identified OSA as an independent risk factor for the development of HF [47], with more impact in men than in women [48]. Prospective data from the Wisconsin Sleep Cohort Study in a cohort of 1131 adults aged 30–60 and followed for 24 years show a 2.6-fold increase in the incidence of coronary heart disease and (self-reported) HF, after adjustment for age, sex, body mass index, and smoking [49].

Once HF has developed, SDB is common, with prevalence rates of 50–75 % [50, 51] in both HFrEF [52, 53] and HF with preserved ejection fraction (HFpEF) [54, 55], with no difference in prevalence between the two groups [56•]. SDB is also common in acute decompensated HF, with reported prevalence rates of between 44 and 97 % [57, 58].

The prevalence of CSA (including Cheyne-Stokes respiration (CSR)) appears to increase as the symptomatic severity of the HF syndrome increases [50, 54], and the severity of CSA/CSR seems to mirror underlying cardiac dysfunction [59]. Furthermore, CSA is independently associated with a worse prognosis, including increased mortality [60].

Similarly, OSA is independently associated with a worse prognosis in HF [61], even in those who are receiving maximal and optimal HF therapy, including cardiac resynchronisation [62].

Although effective treatment of HF may improve CSA/CSR [63], patients who still manifest CSA/CSR despite maximal and optimal HF therapy, including cardiac resynchronisation [62], have a poorer prognosis than those who do not. In addition, when present, CSA in acute decompensated HF patients is usually severe (apnoea-hypopnoea index (AHI) >30/h) [57] and has been shown to be a predictor of hospital readmission and mortality [64].

## Treatment of SDB in Heart Failure

### Lifestyle Measures

Weight loss significantly reduces AHI in obese patients with OSA [65]. However, patients with HF and OSA are less likely to be obese and the impact in this group is not known.

Patients in whom SDB occurs in a supine sleep position should be counselled regarding *positional therapy*: using a wedge or cushion or sewing a pocket filled with tennis balls on the back of a pyjama shirt can discourage sleep in the supine position. Such an approach appears to work for patients with SDB due to HF [66].

Alcohol, sedatives, narcotics, and muscle relaxants should be avoided, as they may reduce upper airway muscle tone. Drowsy-driving precautions should be reviewed with the patient and advice documented.

### General Medical Optimisation

Optimal medical management is likely to improve SDB. This should include the use of diuretics and disease-modifying therapy such as angiotensin-converting enzyme inhibitors/angiotensin receptor blockers, sacubitril/valsartan, beta-blockers, and aldosterone antagonists. Cardiac resynchronisation therapy (CRT) for patients with heart failure with reduced ejection fraction and a broad QRS complex significantly reduces AHI in CSA (but not OSA) with HF [67].

### Oral Appliances

Oral appliances, worn during sleep and fitted by a dentist, may be used to extend the dimensions of the airway and may be effective in select patients with OSA and retrognathism, particularly if the SDB is mild or positional [68].

### Surgery

Although surgical methods of ameliorating SDB have not been specifically tested in HF, there may be a limited role for such intervention in carefully selected cases with OSA and a BMI >35 kg/m^2^ [69]. Upper airway or craniofacial surgical interventions may be an option for SDB treatment; however, they require careful assessment and evaluation by an experienced otolaryngologist.

### Positive Airway Pressure

#### Obstructive Sleep Apnoea

Positive airway pressure (PAP) therapy delivered through a nasal (or nasal-oral) mask stabilises the airway (preventing collapse) and is the standard treatment for SDB associated with daytime sleepiness in the non-HF population [70, 71]. There are a variety of different treatment modalities, including continuous positive airway pressure (CPAP) therapy [72].

An overnight PAP titration study is required to determine the optimal pressure setting that reduces the number of apnoeas/hypopnoeas during sleep, improves hypoxaemia and sleep architecture, and reduces arousals. Potential beneficial cardiovascular effects of CPAP therapy include increased intrathoracic pressure, reduced LV preload and afterload, and reduced transmural cardiac pressure gradients, all of which can ameliorate impaired cardiac function. CPAP therapy improves daytime somnolence and some measures of quality of life and physical vitality scores in patients with OSA but without HF [73].

Adherence with this therapy is highly variable, with average levels ranging from 50 to 80 %, but with around 70 % still regularly using treatment after 5 years [74]. Adherence is positively influenced by patient education, careful selection of a mask that best fits the patient, and supportive management of nasal congestion or dryness.

In a randomised control trial of 55 patients with HF and OSA, nocturnal CPAP therapy for 3 months improved LV ejection fraction (by 5.0 ± 1.0 vs. 1.0 ± 1.4 %, *p* = 0.04) and reduced urinary noradrenalin excretion [75]. Even one night of CPAP therapy lowers systolic blood pressure (126 ± 6 to 116 ± 5 mmHg, *p* = 0.02), reduces heart rate (68 ± 3 to 64 ± 3/min, *p* = 0.007), and improves LV end-systolic diameter (54.5 ± 1.8 to 51.7 ± 1.2 mm, *p* = 0.009) in those with OSA and HF, compared to standard medical therapy [76]. CPAP therapy improves right ventricular function, left ventricular mass, and pulmonary hypertension after 3 months of treatment, and these improvements persisted at 1 year [77]. An observational study (88 patients) of CPAP therapy versus medical therapy for those with HF and moderate-to-severe OSA demonstrated a significantly higher rate of hospitalisation or death in the non-CPAP therapy group (HR 2.03, CI 1.07 to 3.68, *p* = 0.03) compared to those treated with CPAP therapy [78]. Patients who were not compliant with CPAP therapy also had a higher risk of the composite endpoint. Two other large registry studies found similar results [79, 80].

The 2010 Heart Failure Society of America Comprehensive Heart Failure guidelines recommend screening for SDB and CPAP therapy in those with confirmed OSA [81]. The 2013 ACCF/AHA guidelines states that treating OSA with CPAP therapy in patients with HF does have benefit [82].

Further data will emerge from a randomised trial of adaptive servoventilation device in patients with heart failure and reduced ejection fraction and either predominantly OSA or CSA (ADVENT-HF; NCT01128816), which is currently recruiting patients.

#### Central Sleep Apnoea

A number of treatments for CSA/CSR have been studied, including oxygen, carbon dioxide, CPAP therapy, and adaptive servoventilation (ASV).

Although it does not trigger inspiration during central apnoea, CPAP therapy improves CSA/CSR probably by increasing functional residual capacity (and, as a result, oxygen stores), decreasing blood volume in the lungs and upper airway when lying down, and reducing hyperventilation via a direct effect on the parabasal J receptors of the lung. In addition, CPAP therapy reduces preload and afterload and the cardiac transmural pressure and may benefit cardiac function in some patients.

Early small trials of CPAP therapy in CSA with HF demonstrated an improvement in AHI, reduced daytime plasma natriuretic peptide and catecholamine concentrations, and improved LV ejection fraction. A larger randomised controlled trial (the CANPAP study) was designed to evaluate the effect of CPAP therapy on transplant-free survival in patients with CSA and HF [83]. This trial was stopped early after 258 patients had been randomised and followed up for over 2 years: there was no difference in transplant-free survival between CPAP therapy and the optimal medical therapy alone arm. CPAP therapy improved the AHI (−21 ± 16 vs. –2 ± 18/h, *p* < 0.001), LV ejection fraction (2.2 ± 5.4 vs. 0.4 ± 5.3 %, *p* = 0.02), and 6-min walk test distance and reduced plasma noradrenaline concentrations, but this did not translate into improved survival. Post hoc subgroup analysis suggested that there was a survival advantage in those in whom the AHI was suppressed by CPAP therapy to below 15/h, suggesting a possible role for more efficacious ventilatory techniques, such as ASV [84].

ASV has been shown to be the most effective mask-based intervention for controlling (central) SDB in patients with HF [85]. ASV increases inspiratory support during hypopnoea, withdraws support during hyperventilation, provides mandatory breaths during apnoea, and generates background PAP. It is therefore effective in both CSA and OSA and can suppress complex sleep apnoea [86].

In small randomised clinical trials, beneficial effects of ASV treatment of CSA/CSR in HF patients include significant reductions in AHI, N-terminal pro-B-type natriuretic peptide (BNP) concentrations, urinary catecholamine release, and LV end-systolic diameter; increases in 6-min walk distance and LV ejection fraction; and improved New York Heart Association (NYHA) class [87, 88].

Given these beneficial effects, a large randomised controlled trial, SERVE-HF, was undertaken to assess the impact of ASV on hospitalisation, life-saving cardiovascular intervention, or death in those with HF and CSA [89•]. One thousand three hundred twenty-five patients with a LV ejection fraction ≤45 % and moderate-to-severe (predominantly) CSA were enrolled. At 12 months, ASV was highly efficacious at reducing AHI (from a mean of 31.2/h at baseline to 6.6/h). Despite the good control of the CSA, there was no difference in the primary endpoint between the two groups, and there was a higher overall mortality in those treated with ASV (HR for all-cause mortality 1.28, 95 % CI 1.06 to 1.55, *p* = 0.01; HR for cardiovascular mortality 1.34, 95 % CI 1.09 to 1.65, *p* = 0.006). This trial did not find differences in plasma BNP concentration, 6-min walk test, or health-related quality of life between the two randomised groups. Initial results suggest that the excess mortality was driven by an increase in sudden death, with no difference in deaths from pump failure or admissions to hospital with HF decompensation. Various explanations have been proposed: chance, a direct toxic effect of PAP on patients with poor LV function and a low pulmonary capillary wedge pressure, or that CSA may be at least partially adaptive for patients with severe heart failure [11]. Further data will emerge from the ADVENT-HF study (NT01128816). In the meantime, the use of ASV (or other airway pressure therapies) for the treatment of predominantly central sleep apnoea in HF patients with reduced ejection fraction cannot be recommended. For those already on ASV, they should be counselled about the potential risks of continuing with this therapy.

CSA is found in the majority of patients with acute decompensated (as opposed to chronic) HF, is usually severe, and is associated with an increased risk of readmission and mortality [90]. A randomised trial of ASV in this patient group was initiated but was terminated after the results of SERVE-HF became available (CAT-HF; NCT01953874). The results have yet to be published.

Another area of interest is the use of ASV in patients with HFpEF and CSA/CSR. Early results suggest that ASV can improve cardiac diastolic function, improve symptoms, and decrease B-type natriuretic peptide concentrations in such patients [91, 92]. In addition, the proportion of HFpEF patients treated with ASV who were free of cardiac events were significantly higher than those of untreated patients. No adequately powered randomised trial has been undertaken.

### Oxygen Therapy for CSA/CSR

Oxygen therapy for CSA has been the subject of a few small-scale trials. Its use during sleep reduces the severity of CSA/CSR by approximately 50 %, reduces nocturnal norepinephrine levels, and attenuates apnoea-associated hypoxaemia over time frames ranging from 1 night to 1 month, but only one study has reported clinical improvements [93]. A meta-analysis of the results from 97 patients in the CHF-HOT trials demonstrated a decrease in AHI (−11.4 ± 11.0 vs. −0.2 ± 7.6/h, *p* < 0.01) and an improvement in LV ejection fraction (36.1 ± 11.8 to 46.3 ± 16.2 %, *p* = 0.014) in those with severe CSA treated with home oxygen at 3 l/min via an oxygen concentrator, at least out to 12 weeks [94]. There was also an improvement in mean NYHA class, but no overall improvement in ventricular ectopy or plasma catecholamine concentrations. The impact on prognosis is unknown. A meta-analysis of 14 studies concluded that oxygen therapy does reduce overnight desaturation, but prolongs apnoeas and hypopnoeas [95].

### CO_2_ Therapy for CSA/CSR

Administration of carbon dioxide reduces AHI in CSA, but at the expense of hyperventilation and poor sleep quality, and is not used clinically.

## Experimental Therapies for SDB

### Phrenic Nerve Stimulation

Phrenic nerve stimulation is a new approach to the treatment of CSA/CSR, with initial results showing that it may improve central respiratory events by about 50 % [96•, 97]. The device is similar to a pacemaker, with an electrode that stimulates the phrenic nerve via the left pericardiophrenic or right brachiocephalic vein, implanted percutaneously under sedation in the catheter laboratory. The device unilaterally stimulates the phrenic nerve when no impulse is sensed for a pre-determined time period, inducing a breath. A non-randomised study of 57 patients showed a mean reduction of 55 % in AHI over 3 months (49.5 ± 14.6 to 22.4 ± 13.6/h, *p* < 0.0001), with a reduction in arousals and oxygen desaturation index and improved quality of life [97]. Device or procedure-related adverse events occurred in 26 % of patients, predominantly due to lead displacement. A somewhat larger randomised study has completed recruitment to further evaluate the effect of this technology on the reduction in CSA events but is not powered to determine the effect on hospitalisation or mortality (NCT01816776).

### Hypoglossal Nerve Stimulation

For those with OSA, a device which stimulates the hypoglossal nerve in response to apnoea and hypopnoea can be implanted. An uncontrolled study has shown a significant mean reduction of 68 % in AHI over 12 months in those treated with this stimulator [98]. The impact on cardiovascular outcomes is not known.

### Acetazolamide

Two small trials of acetazolamide have been reported to reduce AHI and improve oxygen saturation in HF and CSA, which may be due its respiratory-stimulating properties as well as a diuretic action [99, 100]. A slightly larger (*n* = 85) randomised study addressing the effect of acetazolamide on the severity of SDB in HF is currently being conducted (Predicting Successful Sleep Apnea Treatment With Acetazolamide in Heart Failure Patients (HF-ACZ), NCT01377987). Whether furosemide achieves the same effect is unknown, although a reduction in pulmonary congestion might be expected to lessen CSA by reducing pulmonary J receptor stimulation.

## Conclusions

SDB is associated with frequent episodic exposure to hypoxaemia, sympathetic nervous system activation, intrathoracic pressure swings, and sleep fragmentation, which exert profound effects on the heart and vasculature. SDB is common in patients with heart failure (although it is rarely associated with daytime sleepiness) and is associated with a poor prognosis. Much of the evidence for the benefit of treatment of SDB comes from observational datasets or small randomised trials in non-heart failure patients, but there is some circumstantial evidence to suggest that the diagnosis and treatment of OSA is worthwhile and may improve cardiac function, sympathetic activation, and symptoms in heart failure. This requires confirmation in an appropriately powered outcome study. A recent randomised trial of the treatment of CSA with non-invasive pressure support reported an unexpected increase in cardiovascular mortality, largely driven by an increase in sudden death. The explanation for this is unclear, but currently, there is no therapeutic imperative to diagnose and treat CSA in patients with heart failure.

Further randomised outcome studies (with cardiovascular mortality and hospitalisation endpoints) are required to determine whether mask-based treatment for SDB is appropriate for patients with chronic systolic heart failure and obstructive sleep apnoea, for those with heart failure with preserved ejection fraction, and for those with decompensated heart failure. New therapies for sleep apnoea—such as implantable phrenic nerve stimulators—also require robust assessment. No longer can the surrogate endpoints of improvement in respiratory and sleep metrics be taken as adequate therapeutic outcome measures in patients with heart failure and sleep apnoea.

Once again, heart failure has confounded predictions—tackling abnormal physiology does not necessarily improve the outcome for patients. Such a link needs to be proven in adequately powered randomised trials.
